# Primary Burkitt’s lymphoma of the thyroid without Epstein-Barr virus infection: A case report and literature review

**DOI:** 10.3892/ol.2014.1941

**Published:** 2014-03-05

**Authors:** LIYING ZHANG, LANXIANG GAO, GUANG LIU, LUPING WANG, CHUNWEI XU, LIN LI, YUWANG TIAN, HUIRU FENG, ZHE GUO

**Affiliations:** 1 Department of Pathology, The Military General Hospital of Beijing PLA, Beijing 100700, P.R. China; 2 Department of Nuclear Medicine, The Military General Hospital of Beijing PLA, Beijing 100700, P.R. China

**Keywords:** Epstein-Barr virus, fluorescence *in situ* hybridization, immunoglobulin rearrangement assay, Burkitt’s lymphoma, thyroid

## Abstract

Primary thyroid lymphomas are rare, and the majority are B-cell lymphoma. Primary Burkitt’s lymphoma (BL) of the thyroid is much less common than the other types of lymphoma. The current study presents the case of an eight-year-old male with a mass in the right lobe of the thyroid, which was detected by B-ultrasound. The patient was diagnosed with BL by immunohistochemistry, fluorescence *in situ* hybridization analysis of MYC (8q24) and immunoglobulin rearrangement assays. Furthermore, subsequent positron emission tomography-computed tomography scans revealed no abnormal metabolites in the left lobe of the thyroid or in other parts of the body following surgery. The patient underwent alternate R-B-NHL-BFM-90-A and R-B-NHL-BFM-90-B treatment for four cycles each following the thyroidectomy. The patient is well and remains free of disease recurrence following almost four years follow-up. The present study discusses this rare case of primary BL of the thyroid and presents a review of the literature. This case report provides evidence that the immediate diagnosis and treatment of primary Burkitt’s lymphoma of the thyroid is likely to improve patient outcome.

## Introduction

Primary thyroid lymphoma accounts for only 2–5% of all thyroid tumors (1,2) and 2.5–7% of extranodal lymphomas (3), which predominantly originate from B lymphocytes. The majority of B-cell lymphomas are diffuse large B-cell lymphomas (DLBCL), extranodal marginal zone B-cell mucosa-associated lymphoid tissue lymphomas (MALToma) and follicular lymphomas (FL) (4). Primary Burkitt’s lymphoma (BL) of the thyroid is even less common than the other types of B-cell lymphoma and is a highly aggressive non-Hodgkin’s lymphoma. Burkitt first identified BL as a sarcoma involving the mandible in African children in 1958 (5). However, due to its rarity, little is known concerning the origin, natural history and effective treatment of primary BL of the thyroid. The current study presents a case of BL and a systematic literature review on the clinical presentation and treatment of this rare tumor. To the best of our knowledge, this is only the fourth case of a primary BL of the thyroid to be reported in the English literature (6–8). Patient provided written informed consent.

## Case report

**Figure 1:**
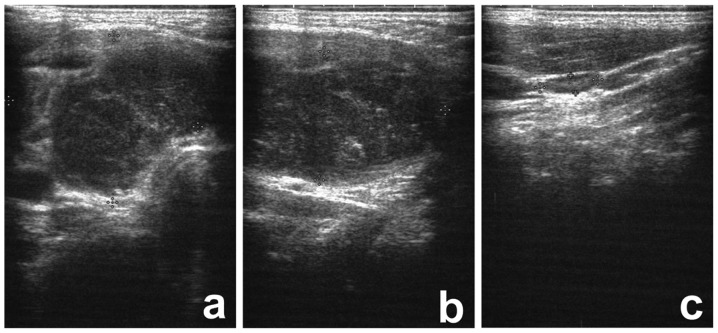
B-ultrasound scan. A mass was detected in the thyroid tissue with (A) the short axis measuring 31.2×26.2 mm and (B) the long axis measuring 39.9×20.3 mm. (C) The surrounding lymph nodes were normal and the largest measured 9.6×2.9 mm in size.

**Figure 2:**
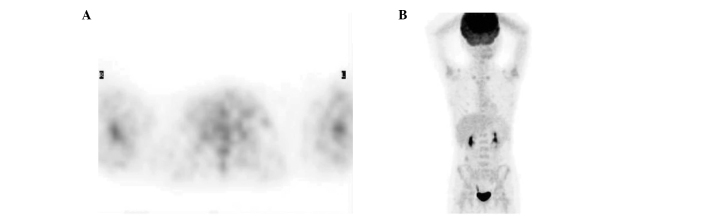
Positron emission tomography-computed tomography following thyroidectomy showing no hypermetabolic nodes in (A) the cervical region and (B) other parts of the body.

**Figure 3:**
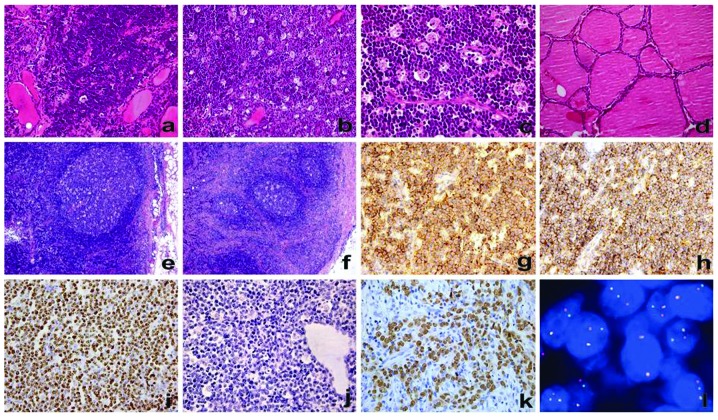
Histopathological observations with hematoxylin and eosin staining. (A) Low magnification showing a diffuse infiltration of atypical lymphoid cells in the thyroid gland (magnification, ×200) and (B) the ‘starry sky’ histology pattern within the tumor tissues (magnification, ×200). (C) Higher magnification showing benign tissue cells engulfing apoptotic bodies (magnification, ×400). (D) Lack of morphological features of Hashimoto’s thyroiditis in the background thyroid gland, including infiltrating lymphocytes, lymphoid follicle formation, stromal fibrosis, eosinophilic change of the epithelial cells or squamous metaplasia (magnification, ×200). (E and F) Reactive lymphoid tissue hyperplasia in the two lymph nodes surrounding the tumor, but not in the neoplastic lesions (magnification, ×100). (G–I) Immunohistochemical staining for the expression of CD20, CD10 and Ki-67 in the neoplastic cells, observed using anti-CD20, anti-CD10 and Ki-67 antibodies with slight hematoxylin counterstain. The positive immunohistochemical signals are brown-yellow, and strong and diffuse (G) anti-CD20 and (H) anti-CD10 antibody immunoreactivity and (I) a high Ki-67 proliferation index (>95%) are apparent in the neoplastic cells (magnification, ×400). (J and K) EBER *in situ* hybridization is (J) negative in the neoplastic cells and (K) positive in the nasopharyngeal carcinoma tissue, which was used as a positive control (EBER digoxin-labeled probe; magnification, ×400). (L) Fluorescence *in situ* hybridization of the MYC (8q24) gene identifying chromosomal translocation in the neoplastic cells (DAPI; magnification, ×1,000). In total, ~90% of the neoplastic cells exhibited visible red and green signal separation, with one yellow fusion signal and two separated red and green signals observed in the majority of the neoplastic cells. (C-MYC break-apart probe). CD, cluster of differentiation; EBER, Epstein-Barr virus-encoded small RNA.

**Figure 4:**
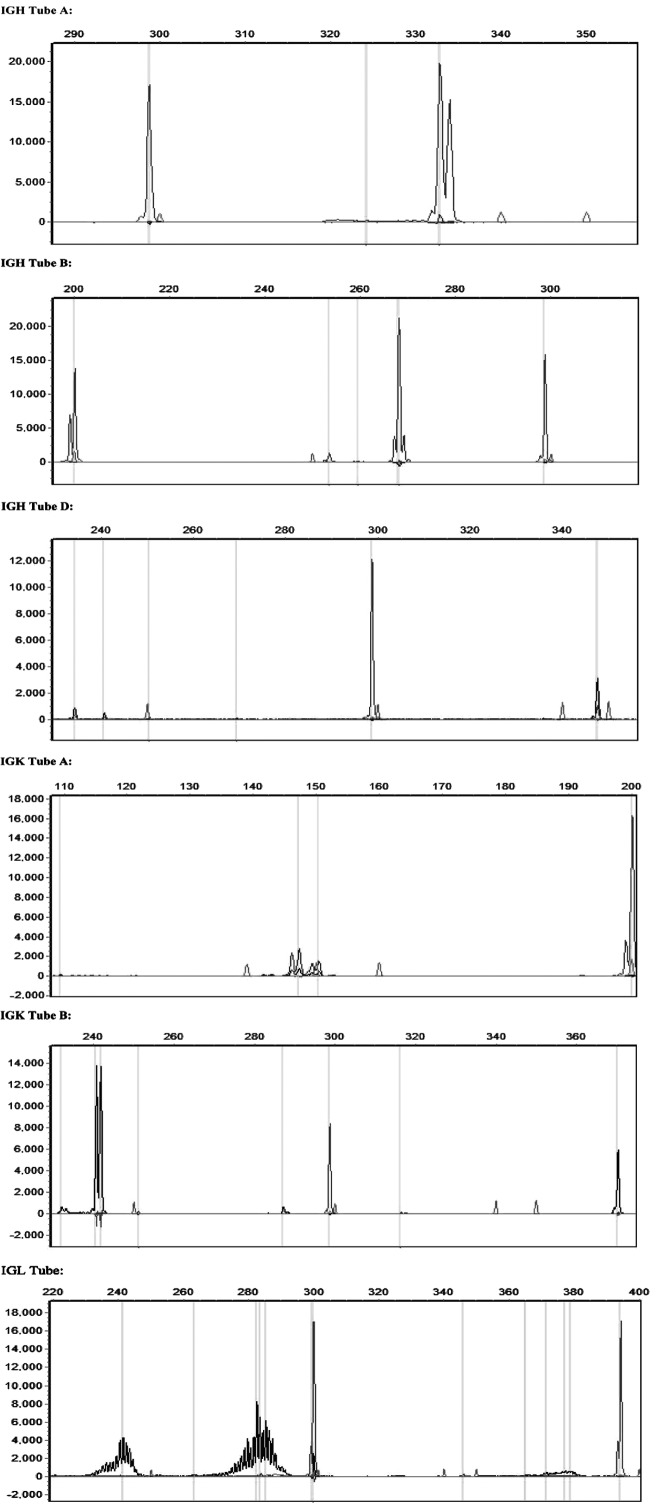
Immunoglobulin gene rearrangement assays. The results showed that IgH-A and -B and IgK-B were positive for gene rearrangements, whereas IgH-D, IgK-A and IgL were negative for gene rearrangements (V_H_ − FR1 + JH consensus for IgH-A; V_H_ − FR2 + JH consensus for IgH-B; D_H_ + JH consensus for IgH-D; Vκ + Jκ for IgK-A; Vκ − Kde and intron Kde for IgK-B; and Vλ + Jλ for IgL; Biomed-2 PCR Kit). IG, immunoglobulin.

An eight-year-old male presented with a mass in the right anterior neck that had been apparent for one week. Upon physical examination, blood pressure was recorded as 100/65 mmHg, heart rate was 80 beats per min, respiratory rate was 20 breaths per min and temperature was 36.1°C. A mass measuring ~4.0 cm in size, which caused difficulty in swallowing, was identified in the right anterior neck. The laboratory test results demonstrated a normal blood count and serum biochemistry, as well as normal levels of electrolytes and carcinoembryonic antigen. In addition, the test results for Epstein-Barr virus (EBV) viral capsid antigens immunoglobulin (Ig)M and IgG, human immunodeficiency virus (HIV) and hepatitis C virus antibodies, hepatitis B antigen and syphilis were negative. Furthermore, the thyroid hormone test results were as follows: Free thyroxine (FT) 4 levels of 11.8 pmol/l (normal range, 9–25 pmol/l); FT3 levels of 4.2 pmol/l (normal range, 3–9 pmol/l); thyroid-stimulating hormone levels of 0.720 μIU/ml (normal range, 0.34–5.60 μIU/ml); anti-thyroglobulin levels of 20 IU/ml (normal range, <115 IU/ml); and anti-thyroid peroxidase levels of 25 IU/ml (normal range, <34 IU/ml). The patient had no significant past medical or family history of disease. A B-mode ultrasound examination revealed a mass measuring 4.0×3.0×2.5 cm in the right lobe of the thyroid (Fig. 1A and B), however, the lymph nodes surrounding the mass were normal (Fig. 1C). The patient underwent a right lobe and isthmus thyroidectomy whereby two lymph nodes were excised simultaneously. Following the surgery, positron emission tomography-computed tomography scans showed normal metabolism in the left lobe of the thyroid and other parts of the body (Fig. 2). The patient’s bone marrow cytology was also normal, however, histological examination revealed diffuse infiltration of atypical lymphocytes and the observation of residual thyroid follicles and necrosis (Fig. 3A). In addition, under low magnification, the ‘starry sky’ histology was observed in certain areas (Fig. 3B). The atypical lymphocytes were medium-sized and consistent, with centrally located nuclei of irregular shape, displaying dispersed and deep basophilic chromatin and scanty cytoplasm. Additionally, certain neoplastic cells were visible, while varying numbers of nucleoli and apoptosis and mitosis were observed. Benign tissue cells engulfing apoptotic bodies were also observed under high magnification (Fig. 3C), however, the isthmus of the thyroid was not infiltrated by the neoplastic cells. No reactive lymphocyte infiltration or fibrosis was identified in the stroma of the thyroid, and no oxyphilic change or squamous metaplasia was observed in the epithelial cells of the background thyroid tissues (Fig. 3D). The only change in the two lymph nodes that were simultaneously excised, was the presence of reactive hyperplasia of the lymphoid follicles (Fig. 3E and F). Immunohistochemical staining was then performed with the primary antibodies shown in Table I (Zymed Corporation, Inc., San Francisco, CA, USA; Santa Cruz Biotechnology, Inc., Santa Cruz, CA, USA). The results showed that the neoplastic cells were diffusely positive for cluster of differentiation (CD)20 (Fig. 3G) and CD10 (Fig. 3H), marginally positive for CD38, CD43 and B-cell lymphoma (Bcl)-6, but negative for Bcl-2 and terminal deoxynucleotidyl transferase (TDT). In addition, CD3 and CD5 stained the background T cells, and the Ki-67 proliferation index was >95% (Fig. 3I). Analysis using an EBV-encoded small RNA (EBER) digoxin-labeled probe (PanPath B.V., Budel, Netherlands) was performed and revealed a negative result (Fig. 3J), however, positive nuclei were observed in the nasopharyngeal carcinoma tissue, which was used as the positive control (Fig. 3K). Analysis using the C-MYC break-apart detection probe (Guangzhou LBP Medical Science Technology Co., Ltd., Guangzhou, China) was also performed and the results revealed that ~90% of the neoplastic cells exhibited red and green signal separation, which indicated that chromosome breakage and translocation of the MYC gene had occurred in the neoplastic cells (Fig. 3L). Immunoglobulin gene rearrangement assays were performed according to instructions of the Biomed-2 Polymerase Chain Reaction kit (Invivoscribe technologies, Inc., San Diego, CA, USA), followed by capillary electrophoresis, which was analyzed using Genemarker^®^ v1.5. software (SoftGenetics, LLC, State College, PA, USA). Positive gene arrangements of IgH and IgK were observed in the tumor tissues, however, no positive gene rearrangements were observed for IgL (Fig. 4). Consequently, the patient was diagnosed with primary BL of the thyroid and underwent alternate R-B-NHL-BFM-90-A and R-B-NHL-BFM-90-B treatment, for four cycles each. The two regimens, including the dose and duration of chemotherapy, are described in Table II. After almost four years of follow-up, the patient appears well and remains free of disease.

## Discussion

Primary lymphoma of the thyroid accounts for only 2–5% of thyroid malignancies and ~5% of extranodal lymphomas. Primary thyroid lymphoma usually occurs in older individuals (mean age, 65 years) and is significantly more common in females than in males. Pediatric head and neck tumors commonly occur in the thyroid between the ages of seven and 13 years (9). Lymphomas of the head and neck in children are predominantly non-Hodgkin’s lymphomas (10) and the majority of these are B-cell lymphomas, which include aggressive DLBCL, MALToma, FL and BL (2,6). BL is significantly more aggressive than the other types of non-Hodgkin’s lymphoma and was first identified in 1958 in African children. According to the World Health Organization (11), BL can be classified into the following three subtypes: Endemic, sporadic and immunodeficiency-associated BL. Endemic BL occurs in equatorial Africa and is one of the most common types of childhood malignancy. Sporadic BL occurs worldwide predominantly in children and young individuals, and immunodeficiency-associated BL usually exhibits a correlation with HIV infection. BL primarily involves extranodal sites, the most common of which are the jaw and facial bones (orbital), the terminal ileum, jejunum, omentum, ovaries, kidneys and breasts. At present, only three cases of primary BL of the thyroid have been reported in the English literature (6–8).

Pediatric lymphoma of the head and neck is likely to present a significant diagnostic problem, particularly in cases where the histological analysis indicates a non-Hodgkin’s lymphoma and the initial site of involvement is extranodal (10). Pediatric lymphoma accounts for ~8.1% of chronic cervical lymphadenopathy in children (12). Furthermore, lymphoma of the thyroid is likely to be derived from persistent low-grade MALToma and to frequently coexist with autoimmune thyroiditis in which the majority of infiltrating cells are of T helper 1 cell origin. This indicates a morphological progression from chronic lymphocytic thyroiditis to low-grade MALToma and ultimately, to high-grade large-cell lymphoma (13). Hashimoto’s thyroiditis (HT), which was first identified by Hashimoto in 1912, is an autoimmune inflammation of the thyroid that commonly affects middle-aged females (14). The histological features of HT include the diffuse infiltration of lymphoid cells (usually with the formation of lymphoid follicles) and varying degrees of fibrosis, oxyphilic change or squamous metaplasia in the epithelial cells. When the presence of focal lymphocytic infiltration is assumed to be an adequate criterion for the diagnosis of autoimmune thyroiditis, the incidence appears to be as high as 16–23% in elderly females. Evidence also exists that indicates a correlation between primary B-cell lymphoma of the thyroid and HT, Sjögren syndrome and rheumatoid arthritis (15,16). The present case of BL, presenting in the thyroid of an eight-year-old male, contradicts the typical age and gender of onset for HT. Morphologically, no lymphocyte infiltration or fibrosis were identified in the stroma of the residual and surrounding thyroid tissues, and no oxyphilic change or squamous metaplasia were observed in the epithelial cells of the background thyroid tissues. In addition, the structures of the two lymph nodes surrounding the mass were normal. Therefore, it may be hypothesized that the pathogenesis of this case of BL did not originate from chronic thyroiditis or the surrounding lymph nodes.

The histological characteristics of BL include the following: Medium-sized and consistent atypical cells with basophilic cytoplasm and small nucleoli; observation of apoptosis and mitosis; the ‘starry sky’ phenomenon; a unique immunophenotype of atypical cells with the expression of IgM, CD10, CD38, CD43 and Bcl-6; the expression of the B-cell-associated antigens (CD20, CD19 and CD22); a Ki-67 proliferation index of ~100% (17); and usually no expression of Bcl-2 and TDT. In the present case, the immunophenotype of the atypical lymphoid cells was consistent with these features.

The MYC gene, a proto-oncogene located at 8q24, is an important member of the *MYC* gene family, which is involved in transcriptional regulation, cell proliferation, growth, differentiation, apoptosis and angiogenesis, and has been associated with the development of a variety of tumors (18). The vast majority of BL cases exhibit the MYC gene translocation, which predominantly occurs in the t(8; 14)(q24; q32) chromosomal region and less frequently in the t(8; 22) chromosomal region (17). BL often exhibits rearrangement of the immunoglobulin heavy chain and light chains, and a translocation between the MYC gene and the immunoglobulin gene loci. The most common translocation is between the MYC gene and the high-activity region of Ig, resulting in the formation of a high-activity transcription rearrangement zone. This subsequently initiates MYC gene transcription and the consequent expression of MYC, which enhances the expression of malignant cells, ultimately resulting in tumorigenesis. Dave *et al* (19) determined that abnormal MYC gene expression is a sign of BL, which indicates that abnormalities in the MYC gene have become a genetic trait of BL and significant for its clinical diagnosis. In the present case, the Ig heavy chain and κ light chains exhibited rearrangement, which confirmed that this case of BL was monoclonal. Additionally, a MYC gene translocation was detected in the majority of the neoplastic cells, which further confirmed the diagnosis of BL.

EBV infection and an inherited immunodeficiency state are considered to be involved in the pathogenesis of lymphoma (10). In addition, it has been confirmed that the occurrence of BL is associated with viral infections, particularly EBV infection. However, the EBV detection rates in different subtypes of BL also vary (20). EBV infection is detected in the vast majority of endemic BL and ~30% of sporadic BL. In addition, the EBV detection rate may marginally differ between BL patients of different regions; for example, a group of Brazilian studies have shown that the EBV detection rates vary between the different regions of Brazil, with the lowest rates observed in the southern regions and the highest rates observed in the northern regions. In addition to EBV infection, BL has also been associated with Kaposi’s sarcoma-associated herpes virus and HIV. In the present case of sporadic BL, the EBER fluorescence *in situ* hybridization detection results were negative, the serological results exhibited no evidence of HIV infection and the patient had no organ transplantation history. Therefore, the probable cause of this case of BL was not associated with EBV or an inherited immunodeficiency state.

BL must be morphologically distinguished from DLBCL, MALToma and B-cell lymphoblastic lymphoma/leukemia. Under normal circumstances, the majority of DLBCL cases are easy to distinguish from BL, although DLBCL may appear morphologically similar to BL, with the appearance of medium-sized cells, the ‘starry sky’ phenomenon, adhesion growth and a high proliferation index. However, as the treatment for these two diseases is different, the identification of DLBCL and BL has important clinical significance (17,21). The detection of a MYC gene translocation is an important method used to identify them, however, the MYC gene rearrangement is not unique to BL and may also occur in certain cases of DLBCL (17). At present, the application of immunohistochemistry is considered to be the most effective and economical method of detection, as the positive results for CD20, CD10 and Bcl-6, and the negative result for Blc-2, together with the high Ki-67 proliferation index (usually >95%), are able to determine a definitive diagnosis for the majority of BL cases. Extranodal marginal zone B-cell MALToma is the most common type of primary thyroid lymphoma, and can be morphologically differentiated from BL. MALT is composed of heterogeous small B cells, which include centrocyte-like cells, monocyte-like B cells, small B lymphocytes and immunoblastic-like cells. In addition, plasma cell differentiation is usually observed, often with the formation of lymphoid follicles and lymphoepithelial lesions. In addition, MALToma does not express CD10 or Bcl-6 and the Ki-67 proliferation index is much lower than that observed in BL. B-cell lymphoblastic lymphoma/leukemia predominantly occurs in children and mitosis is easily observed. Occasionally, it is difficult to differentiate B-cell lymphoblastic lymphoma/leukemia from BL according to morphology, however, immunohistochemistry is useful for distinguishing between them, as the former expresses TDT whereas BL does not.

In conclusion, the current study presents a case of sporadic primary BL of the thyroid occurring in an eight-year-old male, which exhibited the typical morphological features and immunophenotype of BL. This case was also found to exhibit a MYC gene translocation, but was not associated with EBV infection. The follow-up examinations subsequent to the surgical treatment and systemic chemotherapy have since shown that the patient is well and remains free of disease recurrence. Therefore, the immediate diagnosis and timely initiation of chemotherapy may provide a complete response, with prolonged progression-free survival, in patients with BL.

## Figures and Tables

**Table I:** Primary antibodies used for immunohistochemical staining.

Antibody	Clone	Dilution	Corporation purchased from
CD20	L-26	1:100	Zymed Laboratories, Inc.
CD10	56C6	1:100	Zymed Laboratories, Inc.
CD3	PS1	1:100	Zymed Laboratories, Inc.
CD5	SP19	1:100	Zymed Laboratories, Inc.
CD43	MT1	1:100	Zymed Laboratories, Inc.
CD38	SPC32	1:100	Zymed Laboratories, Inc.
TDT	SEN28	1:100	Zymed Laboratories, Inc.
Ki-67	K-2	1:100	Zymed Laboratories, Inc.
Bcl-2	C-2	1:100	Santa Cruz Biotechnology, Inc.
Bcl-6	N-3	1:100	Santa Cruz Biotechnology, Inc.

CD, cluster of differentiation; TDT, terminal deoxynucleotidyl transferase; Bcl, B-cell lymphoma.

**Table II:** Details of the R-B-NHL-BFM-90 A and R-B-NHL-BFM-90 B regimens.

Regimen	Dosage, mg/m^2^	Administration	Date
R-B-NHL-BFM-90 A			
Rituximab	375	i.v.	Prior to D1
Dexamethasone	10	po/i.v.	D1–5
Isophosphamide	800	i.v.	D1–5
Methotrexate	500	24 h i.v.	D1
Adriamycin	150	i.v. (every 12 h)	D4 and 5
Etoposide	100	1 h i.v.	D4 and 5
R-B-NHL-BFM-90 B			
Dexamethasone	10	po/i.v.	D1–5
Cyclophosphamide	200	i.v.	D1–5
Methotrexate	500	24 h i.v.	D1
Adriamycin	25	i.v.	D4 and 5

i.v., intravenous; po, orally; D, day.
